# Supplementary material for: Controllability and Robustness of Functional and Structural Connectomic Networks in Glioma Patients
Source: Cancers (Basel). 2023 May 11;15(10):2714. doi: 10.3390/cancers15102714 (PMC10216571; doi:10.3390/cancers15102714)
Supplement: Supplementary file 1 [file cancers-15-02714-s001.zip › Supplement S1.pdf]

S1: MRI Data Acquisition, Preprocessing and Generation of connectivity matrices - a detailed description

From: Jütten K, Weninger L, Mainz V, Gauggel S, Binkofski F, Wiesmann M, et al. Dissociation of structural and functional connectomic coherence in glioma patients. Scientific Reports. 2021;11(1):16790

“Preprocessing

Diffusion acquisitions were corrected for susceptibility-induced correction with FSL TOPUP as described in (Andersson et al., 2003), and for eddy currents and motion artifacts with FSL EDDY (Andersson & Sotiropoulos, 2016). In five cases, in which the reverse-phase encoded image was corrupted, only FSL EDDY was applied. The T1 image, excluding the tumor, was segmented into WM, gray matter and CSF using FSL FAST. The tissue segmentation, as well as the Brainnetome parcellation (Fan et al., 2016) were transformed into diffusion space using s symmetric diffeomorphic image registration of T1 image and diffusion acquisition (Avants et al., 2008). To distinguish between diffusion anisotropy loss due to fiber degradation and signal loss caused by free water compartments, a free water correction was applied as described elsewhere (Leon Weninger et al., 2020; L. Weninger et al., 2020).

Probabilistic diffusion tractography was performed in native diffusion space using anatomical constraints of the transformed tissue segmentation map. Fiber orientations were obtained with constrained spherical deconvolution (Tournier et al., 2007). Using the obtained fiber orientation distribution function, probabilistic tractography, as implemented in Dipy (Garyfallidis et al., 2014) was performed with a step size of 0.5 mm and a maximum angle between subsequent steps of 30°. Tracking seed point were set to the boundary between gray matter and WM using 3x3x3 seed points per voxel. Tracking was terminated if the FA value was below 0.15 or when the WM boundary was reached. All streamlines that did not terminate in gray matter or where the final length was less than 2 mm were discarded. The remaining streamlines were partitioned into fiber tracts depending on the gray matter start- and end regions as defined in the Brainnetome Atlas (Fan et al., 2016).

Functional preprocessing was performed using SPM12 (Friston et al., 2007; Penny et al., 2006) as implemented in Matlab 9.3 (The MathWorks, 2017). A detailed description of the image preprocessing protocol can be found in previous studies (Jütten et al., 2020; Jütten et al., 2019). In brief, tumor lesions were segmented semi-automatically using the ITK-SNAP software version 3.4.0 (Yushkevich et al., 2006)

(http://www.itksnap.org/pmwiki/pmwiki.php?n=Downloads.SNAP3) and included perifocal T1 hypo- and T2-FLAIR hyperintensities for gliomas grade II-III, as well as T1 hypointensities and contrast-enhancing tumor for glioblastomas. Then, functional images were realigned to the mean functional volume, unwarped and coregistrated to the structural image. Structural and functional images were normalized (including a binary tumor mask in case of patients' data), and functional images were smoothed with a 5 mm FWHM Gaussian kernel. Then, functional images were slice-time corrected and movement-related time series were regressed out with ICA-AROMA (Pruim et al., 2015). Data were high-pass filtered (>0.01 Hz) and parcellated into a set of 246 predefined anatomical brain regions using the Brainnetome Atlas. A list of included left- and right-hemispheric brain regions can be found here (https://atlas.brainnetome.org/download.html).

Whole-brain and DMN SC and FC analyses

To investigate differences in whole-brain distant (contralesional), local (ipsilesional) and interhemispheric SC and FC between patient groups and controls, individual subjects' time-courses were extracted from parcellated atlas regions, which were used as ROIs. For quantification of SC, the edge-weight (EW) between all pairs of ROIs was determined, resulting in a 246 x 246 SC matrix. EW was defined as the number of fiber connections between the two ROIs divided by the mean number of fibers originating or ending within these two ROIs. In addition, mean fractional anisotropy (FA) values were computed for all fibers connecting each ROI with one another, also resulting in a 246 x 246 SC matrix. With regard to FC, all ROIs' mean time-series were computed and cross-correlated within each subject, resulting in a 246x 246 FC matrix. Correlations were Fisher z-transformed. Only those ROIs interconnected by at least one streamline in controls were considered for further analyses. This included 619 left-hemispheric, 637 right-hemispheric, and 92 interhemispheric ROI connections (*Figure 2a*). Based on these remaining ROIs, contra- and ipsi-lesional, as well as interhemispheric means of SC measures (EW, FA) and FC were computed and analyzed for differences between patient groups and controls.

To investigate DMN specific connectivity, Brainnetome Atlas structures comprising the anterior cingulate cortex, posterior cingulate cortex, medial temporal gyrus and inferior parietal lobule were chosen as ROIs and DMN-EW, DMN-FA and DMN-FC were computed" (Jütten et al., 2021, pp. 3-4**).** "Within these DMN hub regions, mean contralesional, ipsilesional, and interhemispheric SC and FC were computed and compared across patient groups and controls" (Jütten et al., 2021, p. 4**).**

References

Andersson, J. L., Skare, S., & Ashburner, J. (2003). How to correct susceptibility distortions in spin-echo echo-planar images: application to diffusion tensor imaging. *Neuroimage, 20*(2), 870-888. https://doi.org/10.1016/S1053-8119(03)00336-7

Andersson, J. L. R., & Sotiropoulos, S. N. (2016). An integrated approach to correction for off-resonance effects and subject movement in diffusion MR imaging. *Neuroimage*, *125*, 1063-1078. https://doi.org/10.1016/j.neuroimage.2015.10.019

Avants, B. B., Epstein, C. L., Grossman, M., & Gee, J. C. (2008). Symmetric diffeomorphic image registration with cross-correlation: evaluating automated labeling of elderly and neurodegenerative brain. *Med Image Anal*, *12*(1), 26-41. https://doi.org/10.1016/j.media.2007.06.004

Fan, L., Li, H., Zhuo, J., Zhang, Y., Wang, J., Chen, L., Yang, Z., Chu, C., Xie, S., Laird, A. R., Fox, P. T., Eickhoff, S. B., Yu, C., & Jiang, T. (2016). The Human Brainnetome Atlas: A New Brain Atlas Based on Connectional Architecture. *Cereb Cortex*, *26*(8), 3508-3526. https://doi.org/10.1093/cercor/bhw157

Friston, K. J., Ashburner, J., Kiebel, S. J., Nichols, T. E., & Penny, W. D. (2007). *Statistical Parametric Mapping: The Analysis of Functional Brain Images*. Academic Press. https://doi.org/http://store.elsevier.com/product.jsp?isbn=9780123725608

Garyfallidis, E., Brett, M., Amirbekian, B., Rokem, A., van der Walt, S., Descoteaux, M., Nimmo-Smith, I., & Dipy, C. (2014). Dipy, a library for the analysis of diffusion MRI data. *Front Neuroinform*, *8*, 8. https://doi.org/10.3389/fninf.2014.00008

Jütten K, Weninger L, Mainz V, Gauggel S, Binkofski F, Wiesmann M, et al. Dissociation of structural and functional connectomic coherence in glioma patients. Scientific Reports. 2021;11(1):16790.

Jütten, K., Mainz, V., Delev, D., Gauggel, S., Binkofski, F., Wiesmann, M., Clusmann, H., & Na, C.-H. (2020). Asymmetric tumor-related alterations of network-specific intrinsic functional connectivity in glioma patients. *n/a*(n/a). https://doi.org/10.1002/hbm.25140

Jütten, K., Mainz, V., Gauggel, S., Patel, H. J., Binkofski, F., Wiesmann, M., Clusmann, H., & Na, C. (2019). Diffusion Tensor Imaging Reveals Microstructural Heterogeneity of Normal-Appearing White Matter and Related Cognitive Dysfunction in Glioma Patients [Original Research]. *9*(536). https://doi.org/10.3389/fonc.2019.00536

Penny, W., Friston, K., Ashburner, J., Kiebel, S., & Nichols, T. (2006). *Statistical Parametric Mapping: The Analysis of Functional Brain Images* (1st Edition ed.). Academic Press.

Pruim, R. H. R., Mennes, M., van Rooij, D., Llera, A., Buitelaar, J. K., & Beckmann, C. F. (2015). ICA-AROMA: A robust ICA-based strategy for removing motion artifacts from fMRI data. *Neuroimage*, *112*, 267-277. https://doi.org/doi.org/10.1016/j.neuroimage.2015.02.064

The MathWorks. (2017). *Matlab2017b*. In (Version 9.3)

Tournier, J. D., Calamante, F., & Connelly, A. (2007). Robust determination of the fibre orientation distribution in diffusion MRI: non-negativity constrained super-resolved spherical deconvolution. *Neuroimage*, *35*(4), 1459-1472. https://doi.org/10.1016/j.neuroimage.2007.02.016

Weninger, L., Koppers, S., Na, C.-H., Juetten, K., & Merhof, D. (2020). Free-Water Correction in Diffusion MRI: A Reliable and Robust Learning Approach. In E. Bonet-Carne, J. Hutter, M. Palombo, M. Pizzolato, F. Sepehrband, & F. Zhang, *Computational Diffusion MRI* Cham.

Weninger, L., Na, C. H., Jutten, K., & Merhof, D. (2020). Analyzing the effects of free water modeling by deep learning on diffusion MRI structural connectivity estimates in glioma patients. *PLoS One*, *15*(9), e0239475. https://doi.org/10.1371/journal.pone.0239475

Yushkevich, P. A., Piven, J., Hazlett, H. C., Smith, R. G., Ho, S., Gee, J. C., & Gerig, G. (2006). User-guided 3D active contour segmentation of anatomical structures: Significantly improved efficiency and reliability. *Neuroimage*, *31*(3), 1116-1128. https://doi.org/doi.org/10.1016/j.neuroimage.2006.01.015
