# Supplementary material for: Controllability and Robustness of Functional and Structural Connectomic Networks in Glioma Patients
Source: Cancers (Basel). 2023 May 11;15(10):2714. doi: 10.3390/cancers15102714 (PMC10216571; doi:10.3390/cancers15102714)
Supplement: Supplementary file 1 [file cancers-15-02714-s001.zip › Supplement S2.pdf]

S2: A detailed list of included DMN ROIs

Lobe	Gyrus	Left and Right Hemisphere	Label ID.L	Label ID.R	Anatomical and modified Cyto-architectonic descriptions	lh.MNI(X,Y,Z)	rh.MNI(X,Y,Z)
**Frontal Lobe**	SFG, superior frontal gyrus	SFG_L(R)_7_6	11	12	*A9m,medial area 9*	-5, 36, 38	6, 38, 35
		SFG_L(R)_7_7	13	14	*A10m, medial area 10*	-8, 56, 15	8, 58, 13
	OrG, Orbital Gyrus	OrG_L(R)_6_1	41	42	*A14m, medial area 14*	-7, 54, -7	6, 47, -7
**Temporal Lobe**	MTG, Middle Temporal Gyrus	MTG_L(R)_4_1	81	82	*A21c, caudal area 21*	-65, -30, -12	65, -29, -13
		MTG_L(R)_4_2	83	84	*A21r, rostral area 21*	-53, 2, -30	51, 6, -32
**Parietal Lobe**	IPL, Inferior Parietal Lobule	IPL_L(R)_6_1	135	136	*A39c, caudal area 39(PGp)*	-34, -80, 29	45, -71, 20
		IPL_L(R)_6_2	137	138	*A39rd, rostrodorsal area 39(Hip3)*	-38, -61, 46	39, -65, 44
		IPL_L(R)_6_5	143	144	*A39rv, rostroventral area 39(PGa)*	-47, -65, 26	53, -54, 25
	Pcun, Precuneus	PCun_L(R)_4_3	151	152	*dmPOS, dorsomedial parietooccipital sulcus(PEr)*	-12, -67, 25	16, -64, 25
		PCun_L(R)_4_4	153	154	*A31, area 31 (Lc1)*	-6, -55, 34	6, -54, 35
**Limbic Lobe**	CG, Cingulate Gyrus	CG_L(R)_7_1	175	176	*A23d, dorsal area 23*	-4, -39, 31	4, -37, 32
		CG_L(R)_7_4	181	182	*A23v, ventral area 23*	-8, -47, 10	9, -44, 11
		CG_L(R)_7_7	187	188	*A32sg, subgenual area 32*	-4, 39, -2	5, 41, 6

Note. Modified table based on the file “BNA_subregions.xlsx“ downloaded from the Brainnetome Atlas homepage (https://atlas.brainnetome.org/download.html.)
